# Complementary Specializations of the Left and Right Sides of the Honeybee Brain

**DOI:** 10.3389/fpsyg.2019.00280

**Published:** 2019-02-14

**Authors:** Lesley J. Rogers, Giorgio Vallortigara

**Affiliations:** ^1^ School of Science and Technology, University of New England, Armidale, NSW, Australia; ^2^ Centre for Mind/Brain Sciences, University of Trento, Rovereto, Italy

**Keywords:** isoamyl acetate, orienting response, lateralization, fight, flight, floral scents, antennae, proboscis extension response

## Abstract

Honeybees show lateral asymmetry in both learning about odors associated with reward and recalling memory of these associations. We have extended this research to show that bees exhibit lateral biases in their initial response to odors: viz., turning toward the source of an odor presented on their right side and turning away from it when presented on their left side. The odors we presented were the main component of the alarm pheromone, isoamyl acetate (IAA), and four floral scents. The significant bias to turn toward IAA odor on the right and away from it on the left is, we argue, a lateralization of the fight-flight response elicited by this pheromone. It contrasts to an absence of any asymmetry in the turning response to an odor of the flowers on which the bees had been feeding prior to testing: to this odor they turned toward when it was presented on either the left or right side. Lemon and orange odors were responded to differently on the left and right sides (toward on the right, away on the left), but no asymmetry was found in responses to rose odor. Our results show that side biases are present even in the initial, orienting response of bees to certain odors.

## Introduction

Honeybees have lateralized responses to olfactory stimuli, shown as a bias of better learning to associate an odor with a food reward *via* use of the right antenna (Letzkus et al., 2006). In bees trained using both antennae, memory recall of an odor-reward association is better *via* stimulation of the right antenna in the shorter term after learning and *via* the left antenna in the longer term after learning (Rogers and Vallortigara, 2008). These asymmetries are matched by left-right asymmetry of neural olfactory coding in the antennal lobes (Rigosi et al., 2015) and asymmetrical gene expression in the left and right sides of the brain (Guo et al., 2016).

Honeybees also have lateralized response to an unconditioned stimulus: by presenting sucrose solution to the left or right antennae and scoring proboscis extension response (PER) Baracchi et al. (2018) showed that the right antenna was more responsive than the left antenna and also that the right antenna was more resistant to habituation of the response than was the left antenna. These asymmetries may, at least in part, depend on a higher number of olfactory receptors on the right antennae of honey bees (Frasnelli et al., 2010a). Response to the odor of killed brood is, however, stronger in honeybees using their left antenna than in those using their right antenna, as shown by electroantennography of hygienic, worker bees (McAfee et al., 2017).

In the current study, we tested lateralization of initial responses of honeybees to floral odors and to the alarm pheromone, isoamyl acetate (IAA). IAA, also referred to as isopentyl acetate, is the main active component of the sting pheromone of the honeybee (Boch et al., 1962), and the function of sting pheromone is to recruit bees from the hive to attack (Free, 1961) or to elicit flight (Wagner and Breed, 2000). The attack response includes, initially, abdomen elevation and pumping (Costa et al., 1996), followed by searching and orienting to the source of the odor, and the bee emits high-pitched buzzing and makes body thrusts, which can lead to stinging. It also increases flight and localization of a moving target (Wagner and Breed, 2000). Consistent with stimulating the fight-flight system, IAA also inhibits foraging behavior (Free et al., 1985; Gong et al., 2017) and it blocks olfactory learning (Urlacher et al., 2010).

As shown by Hunt (2007), IAA primes worker bees for flight and/or fight. *Via* effects on the honeybee’s opioid system, it induces two opposing systems, approach *versus* withdrawal (Nunez et al., 1998). Since, in electroantennographic recordings, the right antenna responds more strongly to IAA than does the left antenna (Anfora et al., 2010), we were interested in investigating the possibility that the detection of IAA *via* the right antenna might elicit approach behavior, in preparation for fighting, whereas the left antenna might elicit withdrawal, as a precursor to flight behavior. In other words, as shown in the vertebrate nervous system (Rogers et al., 2013a), one side of the honeybee brain might control initial approach behavior and the other side control withdrawal behavior (see e.g. Quaranta et al., 2007), bearing in mind that excitatory inputs from each antenna are processed mainly on the ipsilateral side of the brain (Suzuki, 1975). This is at least the case in the antennal lobes, which are the primary olfactory centers, although not at the next level of processing in the mushroom bodies, where after a period of delay the olfactory memory trace becomes bilateral (Sandoz and Menzel, 2001), or in the lateral horn (Carcaud et al., 2015).

Our main hypothesis concerned lateralized turning toward versus away responses to the alarm pheromone, which we compared to turning responses to several familiar and unfamiliar floral odors that suppress the fight/flight response (Nouvian et al., 2016).

## Materials and Methods

Worker honeybees, *Apis mellifera*, were captured while foraging and tested within an hour by presenting odors to the left or the right side of the bee’s head, using a method shown previously to stimulate the left or right antenna (Rogers and Vallortigara, 2008). To assess approach and withdrawal, it was necessary to allow the bees to move relatively free; i.e., not be restricted as they are when harnessed in holders, the method used previously to demonstrate asymmetry of response to odors in bees (e.g., Rogers and Vallortigara, 2008; Baracchi et al., 2018). Therefore, during testing, the bee was tethered using a light thread tied loosely around the body between the thorax and the abdomen and with a length of 20 cm running under the bee’s ventral surface. The end of this thread was anchored to the bench top. This allowed the bee to move by walking or flying in a restricted space. A three-sided enclosure with plain, white walls (40 cm × 40 cm × 30 cm) prevented the bee from receiving patterned visual input frontally or laterally. Odors were presented only when the bee was stationary on the floor of the cage and facing away from the open side of the enclosure. Then, an odor was presented in a droplet at the tip of the needle on a syringe and held at a distance of 1–2 cm to the left or right side of the bee, being sure not to touch the antenna (details in Rogers and Vallortigara, 2008). The first response of the bee was to turn either toward or away from the odor/droplet, and this was recorded. “Turning toward” was recorded if the bee moved its head and antennae, and its whole body, in the direction of the presented droplet. “Turning away” was recorded for head with antennae and whole body turning in a direction away from the droplet. Failure to respond by turning was recorded as “no response” if presentation of odor elicited no immediate turning. In some tests, PER responses were also noted.

The following odors were presented: isoamyl acetate (IAA, 98% Aldrich Chemical Co., lot number 06422AX) at dilutions of 1:300, 1:100, and 1:10; lemon oil (Queen Fine Foods Pty Ltd) diluted 1:300; orange oil (Authentic Oil Co.) undiluted; rose water (Queen Fine Food Pty Ltd) diluted 1:75; and odor of a freshly crushed flower of *Hakea decurrens*. In the latter case, the flower was crushed with an earbud and that was presented in testing. Each odor was presented to a bee five times on the left side and five times on the right side in random order, and intervals between presentations were approximately 60 s. The bees were released after testing.

One set of tests was conducted at the University of New England, Australia, on bees collected as they were feeding on Hakea flowers. These bees were tested with IAA 1:300 dilution, lemon, rose water, and crushed Hakea flowers. There were 12 bees tested on each odor. A second set of tests was conducted in Italy (North-East Italy, near Vicenza, in a garden of flowers) with IAA 1:100 and 1:10 dilution and orange oil. Nine bees were tested on each of these odors.

All comparisons of the scores of left versus right side presentations were made using two-tailed paired t-tests.

## Results

**Figure 1:**
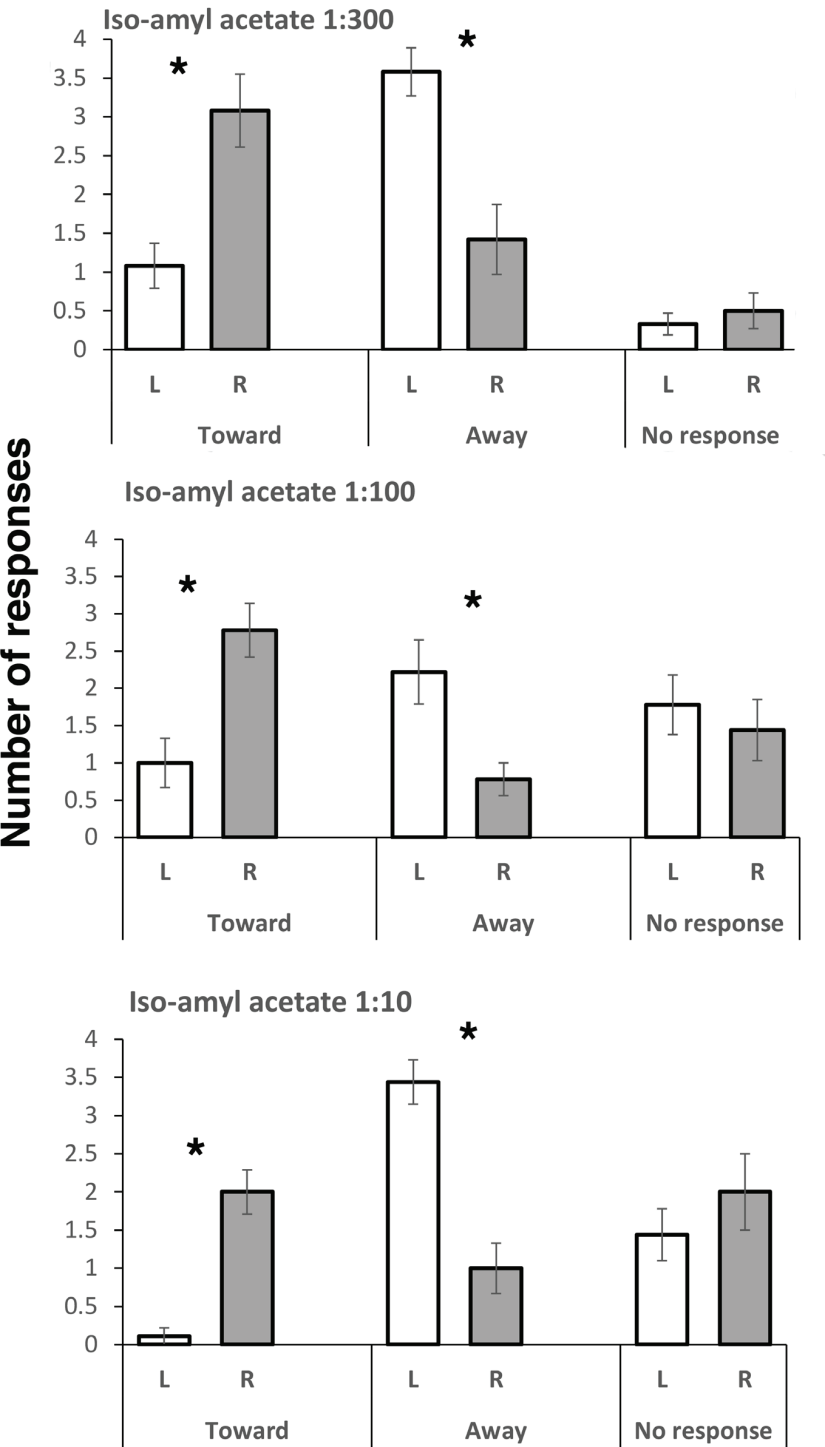
Responses to three concentrations of isoamyl acetate. Means and standard errors are plotted. L and R refer to left antenna and right antenna, respectively. Turning toward and turning away from the presented odor were scored, as well as no response. Asterisks indicate significant differences between left and right antenna. Note that presentation to the right antenna elicits turning toward, whereas presentation to the left antenna elicits turning away, and this is significant at all dilutions of IAA.

Responses to all three concentrations of IAA were similar: viz., turning toward the odor when it was presented on the right side and away from it when it was presented on the left side (Figure 1). For turns toward when presentation of IAA was on the right compared to turning toward when it was presented on the left in the test using 1:300 dilution of IAA, the difference was significant (*t*(11) = 3.388, *p* = 0.006), as it was for the 1:100 dilution (*t*(8) = 3.825, *p* = 0.005) and the 1:10 dilution (*t*(8) = 6.107, *p* = 0.0003). No significant difference in “no responding” scores was found for left versus right comparison at any dose of IAA. However, turning away was more frequent when the odor was presented on the left side compared to the right side (*t*(11) = 3.398, *p* = 0.006 for the 1:300 dilution; *t*(8) = 3.250, *p* = 0.012 for 1:100 dilution; *t*(8) = 5.9330, *p* = 0.0003 for 1:10 dilution). PER responses were scored only in trials with the 1:300 dilution of IAA, and they were rare (mean and standard error of mean, SEM, for the left side were 0.33 ± 0.22, and for the right side, it did not occur).

Body thrusting or pumping, a known response to IAA stimulation (Wagner and Breed, 2000), was scored only for the tests with the 1:300 dilution of IAA, lemon, and rose. Whereas body thrusts occurred in 30% of the bees exposed to IAA, this response did not occur in any of the tests with lemon or rose. There was no significant bias for body pumping to occur more frequently in presentations to the left or right side of the bee (Wilcoxon matched pairs, *p* > 0.05).

**Figure 2:**
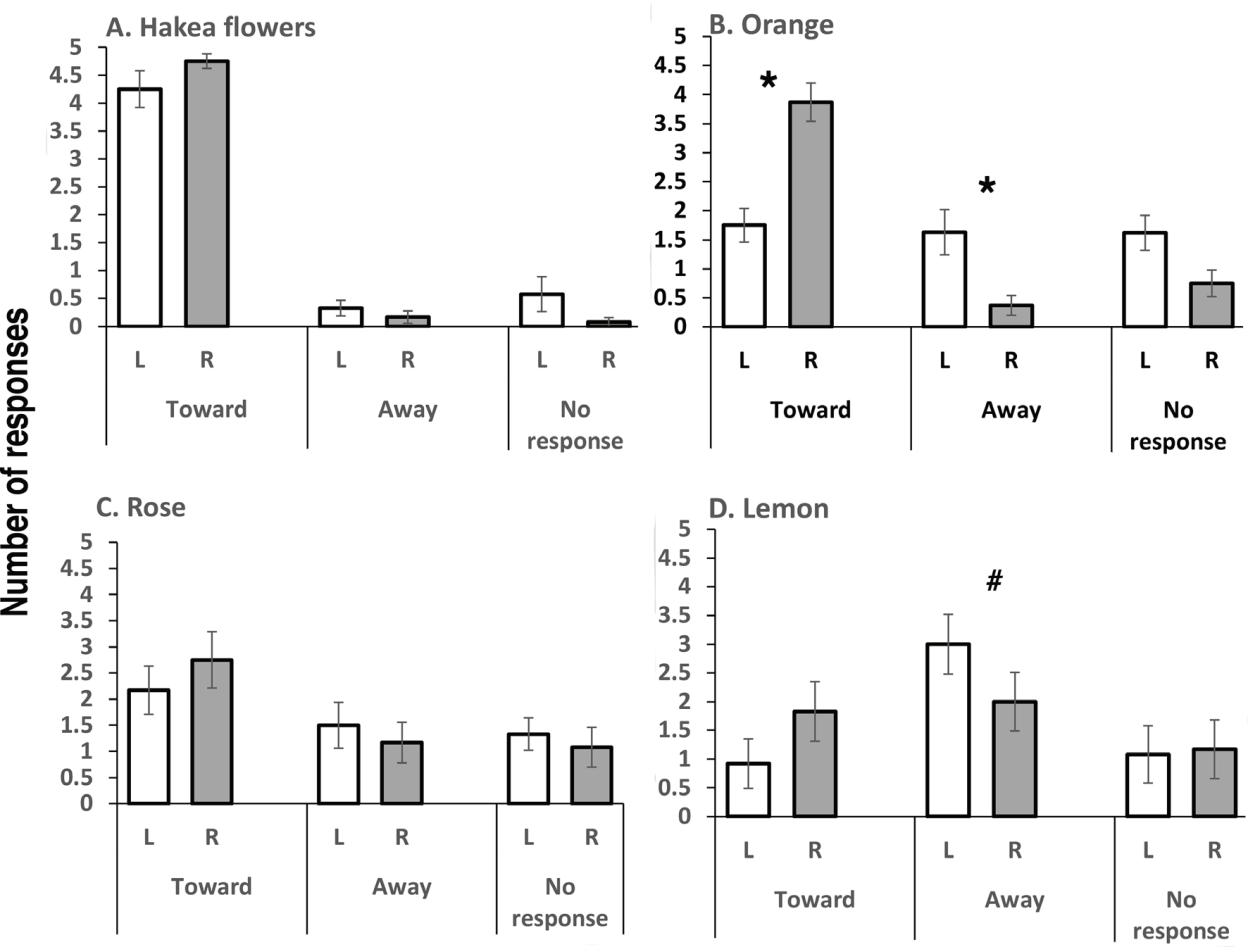
These data are presented in the same manner as in Figure 1. **(A)** Tests with the odor of Hakea flowers: note turning toward for presentations on the left and right and also the few ‘turning away’ and ‘no responses’. **(B)** Orange oil revealed asymmetry: turning toward was significantly higher on presentation of the odor to the right than the left antenna and turning away was significantly higher on presentation to the left than the right antenna. **(C)** Rose water presentation revealed no significant differences between the left and right antennae but note that turning toward was more common than away. **(D)** Lemon elicited more turning toward responses when presented to the right than the left antenna though not significantly, and turning away from the odor was almost significantly higher on presentation to the left than the right antenna (*p* = 0.052, indicated by #). Asterisks indicate significant differences (see text for details).

Bees responded to presentations of the familiar odor of Hakea flowers with consistent turning toward on both the left and right sides (no significant difference between turning toward on the left versus toward on the right side, *t*(11) = 1.4832, *p* = 0.337: Figure 2A). Scores of responding by turning away and those of not responding were very low. Although no lateral asymmetry of turning was present in tests with this odor, PER was more common in tests with this odor than with any other of the tested odors and significantly more so for right-side presentation (mean and SEM; left side: 0.83 + 0.41 and right side: 1.42 ± 0.58; two-tailed t-test, *t*(11) = 2.5483, *p* = 0.027).

Presentation of orange oil revealed asymmetry (Figure 2B): the bees turned toward this odor when it was presented on their right side and did so rarely when it was presented on their left side (*t*(7) = 9.3785, *p* = 0.00001). Presentation on the left side led to either not responding or turning away (comparison of scores for turning away when presentation was on the left versus on the right; *t*(7) = 3.9886, *p* = 0.0053).

Rose water elicited slightly more “turning toward” than “turning away” (Figure 2C), but comparison of scores of turning toward on the left versus the right revealed no significant difference (*t*(11) = 1.1681, *p* = 0.267). A few PER responses occurred (mean and SEM, left side: 0.33 ± 0.33, right side: 0.42 ± 0.34), but there was no significant left-right difference in the PER scores.

In the tests with lemon oil, the bees were more likely to turn away, especially when this odor was presented on the left side (Figure 2D). However, scores for turning away on left-side presentation were not significantly higher than scores for turning away on right-side presentation (*t*(11) = 2.1712, *p* = 0.0526), although there was a trend toward significance. Left-right comparison of scores for turning toward did not reveal a significant difference (*t*(11) = 1.7334, *p* = 0.1109). No PER responses were elicited in trials with lemon oil.

## Discussion

The odors isoamyl acetate (IAA), orange, and (marginally) lemon all revealed significant lateralization of responding: all three of these odors elicited turning toward the source of the odor when it was presented on the bee’s right side and turning away from it when it was presented on the bee’s left side.

The direction of laterality was the same for all doses used to test responses to the alarm pheromone, IAA. At all doses, the bees had higher scores for turning toward the IAA odor when it was presented on their right side and higher scores for turning away when it was presented on their left side. The strengths of these complementary responses were equal at least for the two lower concentrations of IAA. On presentation of the higher concentration of IAA (1:10 dilution), turning away from presentation on the left side was higher than turning toward on the right side.

The bees responded strongly to the natural odor of Hakea flowers, on which they had been feeding immediately prior to being brought to the laboratory for testing, and did so by turning toward the odor when it was presented on their left or on their right side. This result provides a control for the trials with IAA, since it shows that turning direction, on the left side at least, is odor-dependent.

There was also no asymmetry in responding when the bees were tested with rose odor. This result suggests that lateralized responding is odor-dependent or possibly dose-dependent. Further tests with varying doses of floral odorants are needed to determine this.

In the case of IAA, it is possible that turning toward is a precursor to attack, perhaps stimulated by higher responsiveness to IAA by receptors in the right antenna and consistent with the results found by electroantennography (Anfora et al., 2010). Also, higher levels of aggression (indicated by adopting of the C-response body posture) between pairs of bees taken from different hives have been recorded when both bees have only their right antennae intact compared to pairs with only their left antennae intact (Rogers et al., 2013b). Since aggression between bees from different hives is likely to be accompanied by, or triggered by, the release of the alarm pheromone, it seems reasonable to deduce that the bees in our tests with IAA were stimulated to turn toward and attack by inputs from the right antenna to the right side of the brain. The contrasting response of turning away from IAA presented on the left side would, therefore, indicate avoidance of an aggressive encounter.

Presumably, turning toward flower odors is not a precursor to attack but appetitive behavior related to obtaining nectar. In fact, floral odors inhibit aggression in honeybees (Nouvian et al., 2015). Consistent with this, proboscis extension responses (PER) were observed when Hakea odor was presented, significantly more so in response to presentation on the right side than on the left side. This side bias is consistent with specialization of the right antenna to learn to associate an odor with a sugar reward (Letzkus et al., 2006) and to recall short-term memory of this association (Rogers and Vallortigara, 2008).

The response when orange odor was presented was mainly turning toward on the right side (PER not recorded). For lemon odor, it was mainly turning away on the left side and no PER occurred. Since the bees tested in Italy could have had some exposure to orange or other citrus flowers, this might explain their generally positive response stimulated by presentation of odor to their right antenna. By contrast, the bees tested in Australia had had no prior exposure to lemon or other citrus odors, since no species of citrus grow in the high and cold altitude of their location, and this could explain their higher levels of avoidance, especially when lemon was presented on the left side.

We suggest that prior experience with the odors presented may explain the differences in lateralized responses to the floral odors, since we have shown previously that prior experience with an odor associated with reward, and thus already in long-term memory, can cause response competition and absence of antennal laterality in subsequent tests (Frasnelli et al., 2010b).

Lateralization of brain function is characteristic of both vertebrates (Vallortigara and Rogers, 2005; Rogers et al., 2013a; Rogers and Vallortigara, 2015; Vallortigara and Versace, 2017; Ocklenburg and Güntürkün, 2018) and invertebrates (Frasnelli, 2012, 2013, 2014; Rogers et al., 2017). For example, magpies view a model predator (an eagle) with their right eye before they approach it and with their left eye before they take flight from it (Koboroff et al., 2008). This seems to be a pattern of brain asymmetry common to vertebrate species (MacNeilage et al., 2009; Rogers et al., 2013a), including humans (Davidson et al., 1990). Given the fact that the optic nerves decussate almost entirely in species with mainly lateral vision and with very little binocular overlap, this laterality reflects control of approach by the left side of the brain and of withdrawal by the right side of the brain. In other words, the direction of this asymmetry appears to be opposite to that seen in bees, suggesting that the asymmetry in invertebrates is analogous, not homologous, to that in vertebrates.

Some research has been conducted on turning behavior in other invertebrate species although in other contexts than the one we tested. For example, ants, *Temnothorax albipennis*, exploring a branching maze, display a preference to turn leftwards (Hunt et al., 2014) and so do water bugs, *Belostoma flumineum* (Knight et al., 2008). In the case of the ants, the causation of this bias may, at least in part, be explained by the fact that they have a different number of ommatidia in each eye (Hunt et al., 2018).

Although it may not seem immediately obvious why an animal would be stimulated to turn toward an odor on the right and away from it on the left, such occurrence of population-level asymmetries has been modeled by game theoretical analysis when one lateralized individual interacts with another lateralized individual (Ghirlanda and Vallortigara, 2004; Ghirlanda et al., 2008), and it is an evolutionary stable strategy (Vallortigara and Rogers, 2005; see Rogers et al., 2013a,b, 2017 for examples of this in bees). Here, we emphasize that response to alarm pheromone is an important social behavior in bees. We have suggested elsewhere (Vallortigara and Rogers, 2005) that asymmetries may have evolved initially at the individual level as a way to optimize neural circuitry, avoiding duplication of functions and promoting parallel processing (which could have been particularly relevant for animals with a relatively small number of neurons), and that departures from equiprobable distribution of asymmetric forms (or its maintenance) may depend on task demands and interindividual interactions (Frasnelli and Vallortigara, 2018).

Since IAA upregulates the levels of serotonin and dopamine in the honeybee brain (Nouvian et al., 2018), it would be interesting to see whether these neural changes associated with defensive attack are lateralized or, in other words, are controlled predominantly by inputs from the right antenna. Nouvian et al. (2018) have suggested that IAA may not act as an actual stimulus of attack but rather as a modulator of an internal threshold of likelihood to attack. Our results lead us to propose that such a threshold may differ for the left and right antennae and left and right sides of the brain.

It is also possible that visual cues played a role in the laterality that we measured. In the case of IAA, when the odor is detected by the bee on its right side, attention to the visual cues could elicit orientation (turning) to the stimulus to be attacked, whereas, on the left side, the visual cues are ignored and the response is turning away as a precursor to flight. This explanation would be consistent with the honeybee’s known lateralization of attention to visual stimuli. Letzkus et al. (2008) showed that honey bees could be trained to associate a visual cue with a food reward provided that they used their right eye but not when they used their left eye. The defensive behavior of honeybees involves processing of multimodal formation (Nouvian et al., 2016), and different sensory inputs may be assessed in different ways on each side of the brain.

The presence of lateral differences in bees responding to odors should alert researchers of the neuronal aspects of olfactory processing to investigating inputs from the left and right antennae separately, as done by Galizia et al. (1998), although they reported no asymmetry, and by Biswas et al. (2010), who did report asymmetry of neuroligin 1 expression following amputation of the left versus the right antenna. Perhaps because of the Galizia et al. (1998) report, asymmetrical aspects of neural processing of odors have rarely been taken into account (e.g. Wang et al., 2008; Deisig et al., 2010). We suggest that side differences in olfactory responding are important and should be considered in future research.

## Data Availability

The raw data are available on request from lrogers@une.edu.au.

## Ethics Statement

Exempt because the research was on honeybees. No permit needed for the work on bees in Australia or Italy.

## Author Contributions

LR and GV conducted the experiments, calculated the data, and wrote the paper.

### Conflict of Interest Statement

The authors declare that this research was conducted in the absence of any commercial or financial relationships that could be construed as a potential conflict of interest.

## References

[ref1] Anfora G. Frasnelli E. Maccagnani B. Rogers L. J. Vallortigara G. (2010). Behavioural and electrophysiological lateralization in a social (*Apis mellifera*) but not in a non-social (*Osmia cornuta*) species of bee. Behav. Brain Res. 206, 236–239. 10.1016/j.bbr.2009.09.023, PMID: 19766143

[ref2] Baracchi D. Rigosi E. de Brito Sanchez G. Giurfa M. (2018). Lateralization of sucrose responsiveness and non-associative learning in honeybees. Front. Psychol. 9:425. 10.3389/fpsyg.2018.00425 29643828 PMC5883546

[ref3] Biswas S. Reinhard J. Oakeshott J. Russell R. Srinivasan M. V. Claudianos C. (2010). Sensory regulation of neuroligins and neurexin 1 in the honeybee brain. PLoS One 5:e9133. 10.1371/journal.pone.0009133, PMID: 20161754 PMC2817746

[ref4] Boch R. Shearer D. A. Stone B. C. (1962). Identification of iso-amyl acetate as an active component in the sting pheromone of the honeybee. Nature 195, 1018–1020. 10.1038/1951018b0, PMID: 13870346

[ref5] Carcaud J. Giurfa M. Sandoz J.-C. (2015). Differential combinatorial coding of pheromones in two olfactory subsystems of the honey bee brain. J. Neurosci. 35, 4157–4167. 10.1523/JNEUROSCI.0734-14.2015, PMID: 25762663 PMC6605296

[ref6] Costa H. Talora D. C. Palma M. S. Chaud-Netto J. (1996). Chemical communication in *Apis mellifera*: temporal modulation of alarm behaviors. J. Venom. Anim. Toxins 2, 39–45. 10.1590/S0104-79301996000100005

[ref7] Davidson R. J. Ekman C. D. Saron J. A. Senulis J. A. Friesen W. V. (1990). Approach-withdrawal and cerebral asymmetry: emotional expression and brain physiology. J. Exp. Psychol. 58, 330–341. 2319445

[ref8] Deisig N. Giurfa M. Sandoz J. C. (2010). Antennal lobe processing increases separability of odor mixture representations in the honeybee. J. Neurophysiol. 103, 2185–2194. 10.1152/jn.00342.2009, PMID: 20181736

[ref9] Frasnelli E. (2013). Brain and behavioural lateralization in invertebrates. Front. Psychol. 4:939. 10.3389/fpsyg.2013.00939, PMID: 24376433 PMC3859130

[ref10] Frasnelli E. Vallortigara G. Rogers L. J. (2010b). Response competition associated with right-left antennal asymmetries of new and old olfactory memory traces in honeybees. Behav. Brain Res. 209, 36–41. 10.1016/j.bbr.2010.01.014 20085786

[ref11] Frasnelli E. Vallortigara G. (2018). Individual-level and population-level lateralization: two sides of the same coin. Symmetry 10:739. 10.3390/sym10120739

[ref12] Frasnelli E. Vallortigara G. Rogers L. J. (2012). Left-right asymmetries of behavioural and nervous system in invertebrates. Neurosci. Biobehav. Rev. 36, 1273–1291. 10.1016/j.neubiorev.2012.02.006, PMID: 22353424

[ref13] Frasnelli E. Anfora G. Trona F. Tesarolo F. Vallortigara G. (2010a). Morpho-functional asymmetry of the olfactory receptors of the honeybee (*Apis mellifora*). Behav. Brain Res. 209, 221–225. 10.1016/j.bbr.2010.01.046 20138089

[ref14] Frasnelli E. Haase A. Rigosi E. Anfora G. Rogers L. J. Vallortigara G. (2014). The bee as a model to investigate brain and behavioural asymmetries. Insects 5, 120–138. 10.3390/insects5010120, PMID: 26462583 PMC4592634

[ref15] Free J. B. (1961). The stimuli releasing the stinging response of honeybees. Anim. Behav. 9, 193–196. 10.1016/0003-3472(61)90008-2

[ref16] Free J. B. Pickett J. A. Ferguson A. W. Simpkins J. R. Smith M. C. (1985). Repelling foraging honeybees with alarm pheromones. J. Agric. Sci. 105, 255–260. 10.1017/S0021859600056318

[ref17] Galizia C. G. Nägler K. Hölldobler B. Menzel R. (1998). Odour coding is bilaterally symmetrical in the antennal lobes of honeybees (*Apis mellifera*). Eur. J. Neurosci. 10, 2964–2974. 10.1111/j.1460-9568.1998.00303.x, PMID: 9758166

[ref18] Ghirlanda S. Vallortigara G. (2004). The evolution of brain lateralization: a game-theoretical analysis of population structure. Proc. R. Soc. Lond. B 271, 853–857. 10.1098/rspb.2003.2669 PMC1691668 15255105

[ref19] Ghirlanda S. Frasnelli E. Vallortigara G. (2008). Intraspecific competition and coordination in the evolution of lateralization. Philos. Trans. R. Soc. B 364, 861–866. 10.1098/rstb.2008.0227 PMC2666077 19064359

[ref20] Gong Z. Wang C. Dong S. Zhang X. Wang Y. Hu Z. (2017). High concentrations of the alarm pheromone component, isopentyl acetate, reduces foraging and dancing in *Apis mellifera* Ligustica and *Apis cerana* Cerana. J. Insect Behav. 30, 188–198. 10.1007/s10905-017-9606-4

[ref21] Guo Y. Wang Z. Li Y. Wie G. Yuan J. Sun Y. (2016). Lateralization of gene expression in the honeybee brain during olfactory learning. Sci. Rep. 6:34727. 10.1038/srep34727 27703214 PMC5050455

[ref22] Hunt G. J. (2007). Flight and fight: a comparative view of the neurophysiology and genetics of honey bee defensive behaviour. J. Insect Physiol. 53, 399–410. 10.1016/j.jinsphys.2007.01.010, PMID: 17379239 PMC2606975

[ref23] Hunt E. R. O’Shea-Wheller T. Albery G. F. Bridger T. H. Gumn M. Franks N. R. (2014). Ants show a leftward turning bias when exploring unknown nest sites. Biol. Lett. 10:20140945. 10.1098/rsbl.2014.0945, PMID: 25540159 PMC4298197

[ref24] Hunt E. R. Dornan C. Sendova-Franks A. B. Franks N. R. (2018). Asymmetric ommatidia count and behavioural lateralization in the ant *Temnothorax albipennis*. Sci. Rep. 8:5825. 10.1038/s4158-018-23652-4 29643429 PMC5895843

[ref25] Knight S. L. Steelman L. Coffey G. Lucente J. Castillo M. (2008). Evidence of population-level lateralized behaviour in giant water bugs, *Belostoma flumineum* Say (Heroptera: Belostomatidae): T-maze turning is left biased. Behav. Process. 79, 66–69. 10.1016/j.beproc.2008.04.001, PMID: 18490112

[ref26] Koboroff A. Kaplan G. Rogers L. J. (2008). Hemispheric specialization in Australian magpies (*Gymnorhina tibicen)* shown as eye preferences during response io a predator. Brain Res. Bull. 76, 304–306. 10.1016/j.brainresbull.2008.02.015, PMID: 18498946

[ref27] Letzkus P. Boeddeker N. Wood J. T. Zhang S-W. Srinivasan M. V. (2008). Lateralization of visual learning in the honeybee. Biol. Lett. 4, 16–18. 10.1098/rsbl.2007.0466, PMID: 18029300 PMC2412924

[ref28] Letzkus P. Ribi W. A. Wood J. T. Zhu H. Zhang S. W. Srinivasan M. V. (2006). Lateralization of olfaction in the honeybee *Apis mellifora*. Curr. Biol. 16, 1471–1476. 10.1016/j.cub.2006.05.060, PMID: 16860748

[ref29] MacNeilage P. F. Rogers L. J. Vallortigara G. (2009). Origins of the left and right brain. Sci. Am. 301, 60–67. 10.1038/scientificamerican0709-60, PMID: 19555025

[ref30] McAfee A. Collins T. F. Madilao L. F. Foster L. J. (2017). Odorant cues linked to social immunity induce lateralized antenna stimulation in honey bees (*Apis mellifera* L.). Sci. Rep. 7:46171. 10.1038/srep46171 28387332 PMC5384011

[ref31] Nouvian M. Reinhard J. Giurfa M. (2016). The defensive response of the honeybee *Apis mellifera*. J. Exp. Biol. 219, 3505–3517. 10.1242/jeb.143016, PMID: 27852760

[ref32] Nouvian M. Hotier L. Claudianos C. Giurfa M. Reinhard J. (2015). Appetitive floral odours prevent aggression in honeybees. Nat. Commun. 6:10247. 10.1038/nccomms10247 26694599 PMC4703898

[ref33] Nouvian M. Mandal S. Jamme C. Claudianos C. d’Ettorre P. Reinhard J. (2018). Cooperative defence operates by social modulation of biogenic amine levels in the honey bee brain. Proc. R. Soc. B 285:2017265. 10.1098/rspb.2017.2653 PMC5805953 29367399

[ref34] Nunez J. Almeida L. Balderrama N. Giurfa M. (1998). Alarm pheromone induces stress analgesia *via* an opioid system in the honeybee. Physiol. Behav. 63, 75–78. 10.1016/S0031-9384(97)00391-0 9402618

[ref35] Ocklenburg S. Güntürkün O. (2018). The lateralized brain: the neuroscience and evolution of hemispheric asymmetries. London: Academic Press. 10.1080/1357650X.2018.1499749 30016906

[ref36] Quaranta A. Siniscalchi M. Vallortigara G. (2007). Asymmetric tail-wagging responses by dogs to different emotive stimuli. Curr. Biol. 17, R199–R201. 17371755 10.1016/j.cub.2007.02.008

[ref37] Rigosi E. Haase A. Rath L. Anfora G. Vallortigara G. Szyszka P. (2015). Asymmetric neural coding revealed by *in vivo* calcium imaging in the honey bee brain. Proc. R. Soc. B 282:20142571. 10.1098/rspb.2014.2571 PMC4345443 25673679

[ref38] Rogers L. J. Vallortigara G. (2008). From antenna to antenna: lateral shift of olfactory memory recall by honeybees. PLoS One 3:e2340. 10.1371/journal.pone.0002340, PMID: 18523636 PMC2394662

[ref39] Rogers L. J. Vallortigara G. (2015). When and why did brains break symmetry? Symmetry 7, 2181–2194. 10.3390/sym7042181

[ref40] Rogers L. J. Frasnelli E. Versace E. (2017). Lateralized antennal control of aggression and sex differences in red mason bees, *Osmia bicornis*. Sci. Rep. 6:29411. 10.1038/srep29411 PMC4937407 27388686

[ref41] Rogers L. J. Vallortigara G. Andrew R. J. (2013a). Divided brains: the biology and behaviour of brain asymmetries. Cambridge: Cambridge University Press.

[ref42] Rogers L. J. Rigosi E. Frasnelli E. Vallortigara G. (2013b). A right antenna for social behaviour in honeybees. Sci. Rep. 3:2045. 10.1038/srep02045 23807465 PMC3694496

[ref43] Sandoz J-C. Menzel R. (2001). Side-specificity of olfactory learning in the honeybee: generalization between odors and sides. Learn. Mem. 8, 286–294. 10.1101/lm.41401, PMID: 11584076 PMC311384

[ref44] Suzuki H. (1975). Convergence of olfactory inputs from both antennae in the brain of the honeybee. J. Exp. Biol. 62, 11–26. 1151274 10.1242/jeb.62.1.11

[ref45] Urlacher E. Francés B. Giurfa M. Devaud J.-M. (2010). An alarm pheromone modulates appetitive olfactory leaning in the honeybee (*Apis mellifera*). Front. Behav. Neurosci. 4:157. 10.3389/fnbeh.2010.00157 20838475 PMC2936933

[ref46] Vallortigara G. Rogers L. J. (2005). Survival with an asymmetrical brain: advantages and disadvantages of cerebral lateralization. Behav. Brain Sci. 28, 575–614. 10.1017/S0140525X05000105 16209828

[ref47] Vallortigara G. Versace E. (2017). “Laterality at the neural, cognitive, and behavioral levels” in APA handbook of comparative psychology: vol. 1. Basic concepts, methods, neural substrate, and behavior. ed. Call J. (Washington, DC: American Psychological Association), 557–577.

[ref48] Wagner B. R. Breed M. D. (2000). Does honey bee sting alarm pheromone give orientation information to defensive bees? Ann. Entomol. Soc. Am. 93, 1329–1332. 10.1603/0013-8746(2000)093[1329:dhbsap]2.0.co;2

[ref49] Wang S. Sato K. Giurfa M. Zhang S. (2008). Processing of sting pheromone and its components in the antennal lobe of the worker honeybee. J. Insect Physiol. 54, 833–841. 10.1016/j.jinsphys.2008.03.004, PMID: 18455180

